# Discoidin Domain Receptors in Tumor Biology and Immunology: Progression and Challenge

**DOI:** 10.3390/biom15060832

**Published:** 2025-06-07

**Authors:** Heng Zhang, Wenlong Chen, Haitao Zhu, Hsiang-i Tsai

**Affiliations:** Institute of Medical Imaging and Artificial Intelligence, Jiangsu University, Zhenjiang 212013, China; 2212213042@stmail.ujs.edu.cn (H.Z.); 2212313028@stmail.ujs.edu.cn (W.C.)

**Keywords:** DDR1, DDR2, immunity, tumor metastasis

## Abstract

The onset and progression of tumors involve intricate, multifactorial processes. A key component in tumor evolution is the dynamic interaction between cancer cells and the extracellular matrix (ECM). Discoidin Domain Receptors (DDRs), a unique class of collagen-activated receptor tyrosine kinases, serve as critical mediators of cell-ECM communication. Recent studies have uncovered their significant roles in modulating diverse cancer-related processes, including immune responses, cell proliferation, apoptosis, differentiation, metabolic reprogramming, metastasis, and resistance to therapy. This review begins with an overview of the discovery, structural features, and canonical and non-canonical functions of DDRs. It then focuses on the reciprocal regulation between DDRs and collagen in the tumor microenvironment, highlighting how this interplay contributes to cancer progression. Furthermore, we explore the involvement of DDRs in reshaping the tumor immune microenvironment and their influence on various aspects of cancer cell biology. Finally, we summarize the current advances in therapeutic strategies targeting DDRs, offering insights into their potential as biomarkers and drug targets in cancer treatment.

## 1. Introduction

The tumor microenvironment (TME) is a complex network comprising tumor-associated stromal cells, the extracellular matrix (ECM), and various soluble factors. As a key component of the TME, the ECM not only offers structural support to cancer cells, but also dictates various aspects of their biology, including growth, invasion, angiogenesis, and metastasis [1,2,3]. ECM remodeling—encompassing degradation and re-deposition of matrix components—alters local mechanical properties and activates pro-tumor signaling pathways, thus promoting malignant cell behaviors [4]. Increased ECM stiffness in certain tumors hinders T-cell infiltration while concomitantly enhancing macrophage activation and M2-like polarization, thereby supporting tumor progression [5]. ECM remodeling contributes to chemoresistance in various cancer types [6]. These mechanisms underscore the multifaceted role of the ECM in tumor progression and highlight its associated pathways as promising therapeutic targets with substantial research and clinical potential.

Receptor tyrosine kinases (RTKs), as integral members of the tyrosine kinase receptor family, modulate diverse complex biological processes through cellular signal transduction, playing pivotal roles in fundamental life activities, such as cell proliferation, differentiation, and metabolic regulation [7]. Discoidin Domain Receptors (DDRs), a subset of the receptor tyrosine kinase (RTK) group, reside on the cell membrane and are activated by collagen. Recognized as a critical link between the ECM and cancer cells, DDRs facilitate cancer cell proliferation, adhesion, migration, immune evasion, and resistance to therapy [8,9]. Additionally, DDRs contribute to the reorganization of fibrous structures and ECM remodeling [10]. Targeting DDR2 has been shown to enhance T-cell infiltration, improving tumor response to PD-1 immunotherapy [11]. This review primarily addresses the molecular architecture of DDR family members, their biological roles in tumor progression, and potential therapeutic strategies aimed at these receptors.

## 2. The Discovery, Structure, and Function of DDRs

The DDR family was first identified in the early 1980s, with DDR1 being the first discovered in 1993 [12]. Unlike other RTKs activated by soluble growth factors, DDRs are unique in that they are activated by collagens or their fragments [13,14]. DDR2, another member of the DDR family, is also referred to as CCK-2, TYRO10, and TKT [15,16,17].

**Figure 1:**
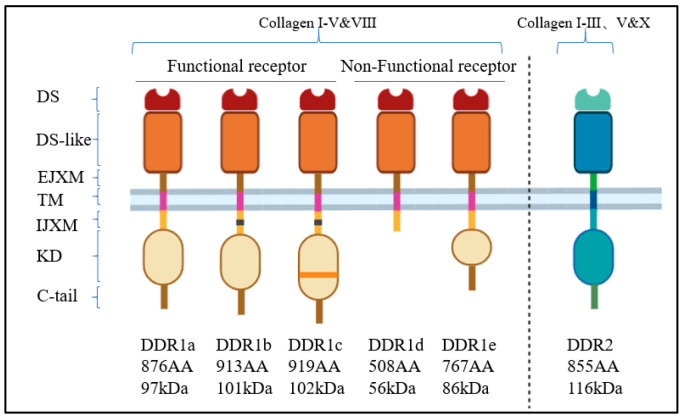
The structural schematics of the five DDR1 isoforms and the unique DDR2.

DDR1 and DDR2, which are encoded by two distinct genes, share notable structural similarities [8]. The DDR family is characterized by several key structural domains: the extracellular collagen-binding DS domain, followed by a discoidin-like domain (DS-like), and an extracellular juxtamembrane domain (EJXM). The EJXM contains approximately 50 amino acid residues in DDR1 and 30 in DDR2. Below this lies the transmembrane domain (TM), followed by the intracellular juxtamembrane domain (IJXM), with roughly 171 amino acid residues in DDR1 and 142 in DDR2. The kinase domain (KD), situated beneath the IJXM, comprises around 300 amino acids. DDR1 features 7 tyrosine residues in the IJXM and 8 in the KD, while DDR2 contains 14 tyrosine residues in the IJXM and 4 in the KD. The sequence concludes with a C-terminal tail [18]. DDR1 exists in five isoforms (DDR1a, DDR1b, DDR1c, DDR1d, DDR1e), with the first three being full-length receptors possessing kinase activity, consisting of 876, 913, and 919 amino acids, respectively. The remaining isoforms lack kinase activity due to truncation or shifting. Collagen binding induces DDR1 to form lateral dimers, which facilitates autophosphorylation between the dimers [19]. DDR2 has a single isoform with kinase activity, composed of 855 amino acids (Figure 1). DDRs can activate multiple signaling pathways, including the MAPK, STAT, and YAP pathways. The expression levels of DDRs are modulated by external factors, such as hypoxia, which has been shown to upregulate DDR2 in vascular smooth muscle cells (VSMC) and DDR1 in pituitary adenomas [20,21].

The expression patterns of DDRs vary depending on the cell and tissue type. DDR1 is predominantly expressed in epithelial cells, while DDR2 is mainly found in mesenchymal cells [22]. Unlike typical RTKs, which are activated by a diverse range of ligands such as growth factors, cytokines, and hormones, DDRs primarily bind to collagen. DDR1 is activated by collagens I–V and VIII, whereas DDR2 interacts with collagens I–III, V, and X [23]. These receptors activate several signaling pathways in tumor cells, including the AKT pathway, the STAT pathway, and the NF-κB signaling pathway [24,25,26].

## 3. The Reciprocal Regulation of DDRs and Collagen in Cancer

Collagen, the primary ligand for DDRs, plays a significant role in various cancer cell behaviors. In hepatocellular carcinoma (HCC), type I collagen promotes cancer cell stemness and metastasis through the CD44/DDR1/YAP axis [24] and the PSD4/ARF6 signaling pathway, respectively [27]. Cleaved type I collagen enhances macropinocytosis and mitochondrial biogenesis in pancreatic cancer cells via the DDR1-NF-κB-NRF2-TFAM axis [26]. In head and neck squamous cell carcinoma (HNSCC) and triple-negative breast cancer (TNBC), type III collagen stimulates DDR1 phosphorylation, activating STAT1 to maintain tumor dormancy [25]. In luminal breast cancer, type IV collagen drives glycolysis in tumor cells through DDR1-mediated MAPK signaling [28]. In pancreatic cancer, type VIII collagen activates PI3K-AKT and NF-κB signaling via DDR1, contributing to drug resistance [29].

In lung adenocarcinoma, type X collagen enhances metastatic potential by promoting FAK phosphorylation through DDR2 activation [30]. In invasive ductal carcinoma (IDC), type I collagen stabilizes the EMT transcription factor Snail via the DDR2-ERK signaling axis, facilitating tumor metastasis [31].

The DDR family engages in bidirectional interaction with collagen, not only being activated by collagen but also modulating its synthesis and alignment [25,32]. Cells expressing DDR1 shed the extracellular domain of DDR1 (DDR1-ECD), which binds to collagen fibers and promotes their ordered alignment, thereby forming a physical barrier that limits immune cell infiltration. This process is independent of DDR1 kinase activity [10].

Thus, DDRs and collagen form a bidirectional regulatory network. The DDR family (DDR1/DDR2) can be activated by various collagen types, influencing diverse cancer cell behaviors. Conversely, DDRs can modulate collagen production and fiber alignment.

## 4. DDRs in Tumorigenesis and Progression

### 4.1. DDRs and the Tumor Immune Microenvironment

Research on the relationship between DDRs and tumor immunity remains limited and warrants further exploration. The following sections discuss the roles of T-cells, neutrophils, and macrophages, as outlined in current studies.

#### 4.1.1. DDRs and T-Cells

**Figure 2:**
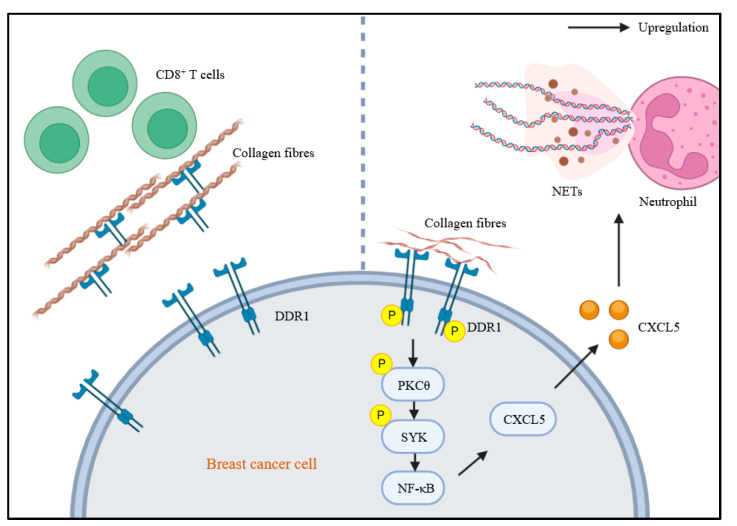
The roles of DDR1 in tumor immunology. The extracellular domain of DDR1 promotes the ordered alignment of collagen fibers upon binding, thereby forming a physical barrier that impedes CD8^+^ T-cell infiltration. Collagen-activated DDR1 upregulates CXCL5 expression via the DDR1/PKCθ/SYK/NF-κB signaling pathway, subsequently driving the formation of neutrophil NETs.

**Figure 3:**
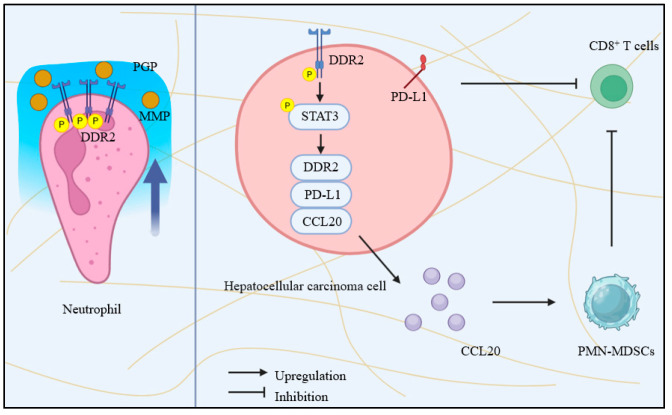
The roles of DDR2 in tumor immunology. In neutrophils, activation of the DDR2 receptor by collagen (brown lines) triggers the release of MMP (orange circles), which degrade the collagen matrix to generate a concentration gradient of PGP (blue halo), thereby enhancing neutrophil chemotaxis and migratory capacity. In HCC, the DDR2/STAT3 positive feedback loop facilitates tumor immune evasion by promoting PD-L1 upregulation and the recruitment of PMN-MDSCs.

DDRs are widely expressed in both cancer and stromal cells, suggesting their involvement in remodeling the tumor microenvironment. DDRs influence T-cell infiltration and distribution within tumor tissues. As mentioned before, DDR1-ECD, rather than its kinase domain, facilitates ECM remodeling and inhibits CD8^+^ T-cell infiltration (Figure 2) [10]. This finding offers new insights into DDR1′s role in the tumor immune microenvironment and may inform the development of future immune therapies targeting DDR1. Activated T-cells enhance migration by upregulating DDR1 expression, which facilitates their adhesion to tissue-localized type I collagen through collagen-binding domains [33,34]. In HCC, collagen-activated DDR2 promotes the transcriptional upregulation of PD-L1, DDR2, and CCL20 via phosphorylation of STAT3. Elevated CCL20 expression recruits polymorphonuclear myeloid-derived suppressor cells (PMN-MDSCs), which, through their surface expression of PD-L1, cooperate to suppress CD8⁺ T-cell-mediated antitumor immune responses (Figure 3) [11,35]. However, evidence directly linking DDRs to T-cell proliferation or cytotoxic functions remains scarce and requires further investigation.

#### 4.1.2. DDRs and Neutrophils

Neutrophils, the most abundant myeloid cells, play a pivotal regulatory role in tumor progression [36]. Activated by type I collagen, DDR1 upregulates CXCL5 in breast cancer cells, promoting the formation of neutrophil extracellular traps (NETs) and Treg infiltration, ultimately driving tumor growth (Figure 2) [37,38]. In neutrophils, collagen initiates a molecular cascade by activating the discoidin domain receptor 2 (DDR2). Upon activation, DDR2 induces the secretion of matrix metalloproteinases (MMPs), which selectively cleave collagen and release peptide fragments enriched with the proline-glycine-proline (PGP) motif. These fragments are subsequently processed by the peptidase prolyl endopeptidase (PE), ultimately generating PGP. The resulting PGP establishes a local concentration gradient that directs neutrophil chemotaxis [39]. The specific role of DDR2 in neutrophils’ regulation of cancer cell biology remains unclear and warrants deeper exploration.

### 4.2. DDRs in Cancer Cell Proliferation and Apoptosis

Unlimited proliferation and resistance to cell death are hallmark characteristics of cancer cells, regulated by various molecular mechanisms. DDR1 plays a pivotal role in promoting cancer cell survival [40,41,42]. Collagen I, secreted by cancer-associated fibroblasts (CAFs), activates DDR1 on pancreatic cancer cells, enhancing macropinocytosis and mitochondrial biogenesis via the DDR1-NF-κB-NRF2 axis, and accelerating cell proliferation in vitro and tumor growth in vivo [26]. In HCC, DDR1 interaction with Solute Carrier Family 1 Member 5 (SLC1A5) prevents lysosomal degradation, stabilizing SLC1A5 and influencing the mTORC1 pathway to promote HCC proliferation [43]. The DDR1/PYK2/ERK signaling cascade activated by collagen also enhances autophagy flux and cell proliferation in pancreatic cancer cells [44]. DDR1 is involved in the formation and progression of KRAS-mutant lung adenocarcinoma, particularly in early stages [45], and its upregulation acts as a compensatory mechanism in KRAS-knockdown cancer cells [46]. In colorectal cancer, low-density lipoprotein receptor-related protein 1 (LRP-1) regulates DDR1 expression via endocytosis, promoting cell cycle progression into the S-phase and enhancing tumor proliferation [47]. Notably, DDR1 exhibits a subtype-dependent regulatory role in breast cancer cell proliferation. In triple-negative breast cancer (TNBC), DDR1 promotes tumor cell proliferation [48]. Conversely, in non-invasive luminal-like pancreatic cancer cells and invasive basal-like breast cancer cells, DDR1 inhibits tumor growth and promotes apoptosis [49,50].

DDR2 has been shown to promote proliferation in several cancers, including melanoma, gastric cancer, HCC, lung cancer [51], breast cancer [52], and ovarian cancer [53]. DDR2 inhibitors suppress HCC proliferation both in vivo and in vitro and promote cancer cell apoptosis [54]. In melanoma, DDR2 stimulates tumor proliferation through the MAP kinase pathway [55], while in lung adenocarcinoma, it enhances cell growth via the focal adhesion kinase (FAK) signaling pathway [30]. In oral squamous cell carcinoma, gene editing of the colon cancer-associated transcript 1 (CCAT1) inhibits the DDR2/ERK/AKT pathway, leading to cell cycle arrest and tumor growth suppression [56]. Additionally, DDR2 regulates T-cell lymphoma apoptosis via the miR-615-5P/DDR2 pathway in lysosomal-associated membrane protein 1 (Circ-LAMP1) modulation [57]. DDR2/STAT1/P27 signaling responds to ECM-derived mechanical forces, inducing cell cycle arrest in breast cancer cells [58]. Collagen-activated DDR2 leads to G0/G1 phase cell cycle arrest and inhibition of fibrosarcoma cell proliferation, exhibiting tumor-suppressive functions [59].

### 4.3. DDRs and Cancer Cell Differentiation

Cancer cell phenotype plasticity is critical for tumor progression, and DDR1 plays a significant role in this process. Under normal physiological conditions, DDR1 is essential for maintaining cellular differentiation and tissue homeostasis [60]. In liver cancer, CD44 binding to DDR1 enhances collagen-induced DDR1 signaling, promoting cancer stemness via the CD44/DDR1/YAP axis [24]. In thyroid cancer, *DDR1* knockdown increases the expression of thyroid differentiation markers (NIS, Tg, TSH, TPO) while decreasing epithelial-mesenchymal transition (EMT) markers and tumor cell stemness [61].

Collagen activation of DDR2 promotes the differentiation of skeletal progenitor cells into osteoblasts [62]. However, the role of DDR2 in tumor differentiation remains unclear, necessitating further investigation.

### 4.4. DDRs and Cancer Cell Metabolism Reprograming

Metabolic reprogramming is another hallmark of cancer [63]. DDR1 activates the PI3K/AKT/PKM2 signaling pathway, facilitating glucose reprogramming in colorectal cancer cells, thereby supporting intracellular homeostasis and cell proliferation [64]. In breast cancer, DDR1 regulates several metabolism-related proteins, including MCT1, MCT4, hexokinase 2, PKM2, NRF-1, and mitochondrial complexes [65]. DDR2 activates the AKT/SNAI1 pathway to enhance hexokinase activity, thereby modulating glycolysis in ovarian cancer cells [66]. Knockdown of *DDR2* in breast cancer cells reduces glutamine and aspartate synthesis [67]. Therefore, DDRs not only regulate molecular signaling pathways but also influence cancer cell behavior through metabolic reprogramming.

### 4.5. DDRs and Tumor Metastasis

Tumor metastasis is a multi-step process, encompassing angiogenesis, EMT, invasion, intravasation, immune modulation, metabolic reprogramming, extravasation, dormancy in micrometastases, pre-metastatic niche formation, and macrometastasis [68]. EMT is a crucial form of phenotype plasticity involved in each metastatic step [69]. DDR1 facilitates cancer cell EMT [70]. Downregulation of ERK signaling by miR-199b-5p inhibits DDR1 expression, suppressing EMT and tumor metastasis in prostate and breast cancer cells [48,71]. Inhibiting DDR1 blocks activation of the Pyk2 and MKK7 signaling pathways in prostate cancer cells, suppressing EMT occurrence [70].

In addition to its role in regulating cancer cell EMT traits, DDR1 has recently been identified as a key regulator of cancer cell dormancy. Dormant cancer cells, characterized by their non-proliferating and cell cycle-arrested state, are increasingly recognized as a critical feature contributing to therapy resistance, recurrence, and metastasis. These dormant cells demonstrate a remarkable ability to adapt to the stresses of the tumor microenvironment, evade immune detection [72], and promote therapeutic resistance [73]. Type III collagen is predominantly produced by cancer-associated fibroblasts (CAFs) but also synthesized by cancer cells. Notably, cancer cell-derived type III collagen autonomously triggers DDR1 activation, which subsequently enhances STAT1 expression. This STAT1 upregulation not only induces cancer cell dormancy but also drives a self-reinforcing cycle by further increasing type III collagen production. The elevated collagen synthesis, primarily originating from cancer cells, disrupts the linear architecture of ECM collagen fibers, thereby amplifying dormancy signals in the tumor microenvironment [25].

DDR1 plays a pivotal role in mediating the adhesion of cancer cells to their microenvironment, a critical factor in cell invasion and metastasis [74]. By stabilizing cell surface E-cadherin, DDR1 enhances intercellular adhesion and facilitates the aggregation of cancer cells [75,76]. Through binding and activating the AKT signaling pathway, DDR1 promotes the migratory and invasive capabilities of lung adenocarcinoma cells [77]. Additionally, DDR1 recruits PSD4 to activate the ARF6-MAPK signaling axis, thereby contributing to the metastasis of HCC cells [27]. In melanoma, the secretion of collagen I by hepatic stellate cells activates DDR1-STAT3 phosphorylation, which exerts a dual effect on cancer cell metastasis. On one hand, DDR1-STAT3 phosphorylation upregulates SOX2, enhancing cancer stemness and supporting the invasion and survival of circulating tumor cells. On the other hand, this phosphorylation event upregulates Mcl-1, which promotes cancer cell proliferation and facilitates the regrowth of circulating cells at secondary sites [78]. Consistent with the seed and soil theory of metastasis, DDR1 expression on cancer cells enhances their ability to localize to tissues rich in collagen III, such as the airway smooth muscle, aiding in tumor colonization [79]. Furthermore, in colorectal cancer, DDR1 activates breakpoint cluster region (BCR) to sustain β-catenin transcriptional activity, a key regulator of cancer cell invasion [80]. In pancreatic cancer, the interaction between DDR1 and Transmembrane-4-L-six-family-1 (TM4SF1) increases the expression of MMP2 and MMP9, enzymes that degrade the basement membrane and disrupt tissue barriers, facilitating cancer cell invasion and metastasis [81]. Similar findings have been observed in osteosarcoma [82]. In gastric cancer, DDR1 knockdown significantly reduces angiogenesis and lymphangiogenesis, as well as lymph node and liver metastasis, highlighting its critical role in metastasis [83]. In the absence of another collagen receptor, integrin, DDR1 can upregulate the MAPK pathway to promote breast cancer cell adhesion to surrounding tissues, independent of collagen receptor signaling [84].

In gastric cancer, DDR2 enhances tumor cell invasiveness and drives EMT by activating mTORC2 and promoting AKT phosphorylation [85]. Increased matrix stiffness leads to upregulation of p300, which subsequently enhances c-Myb acetylation. This, in turn, increases the accumulation of c-Myb and LEF1 at the *DDR2* promoter, resulting in elevated DDR2 expression and facilitating the EMT process in lung cancer cells [86]. In ovarian cancer, DDR2 stabilizes EMT transcription factors, such as Snail1, thus maintaining mesenchymal characteristics and promoting tumor invasion and metastasis [21]. Additionally, DDR2 drives collagen production by CAFs through the upregulation of arginase-1 transcription, further enhancing tumor invasiveness [32]. In gastric cancer, miR-199b-p promotes tumor stemness and metastasis via the DDR2-mTOR-SOX2 axis [87]. In breast cancer, *DDR2* knockdown inhibits tumor cell proliferation and invasiveness [88]. Heat shock protein HSP47 has been shown to bind and stabilize DDR2, thereby promoting tumor invasion [89]. DDR2 also enhances tumor stiffness by reorganizing collagen fibers and recruiting Talin1 and Kindlin2 through the activation of Rap1, facilitating the full activation of integrins bound to collagen. This enhances mechanotransduction and promotes tumor metastasis [90]. Moreover, DDR2 regulates tumor metastasis in ovarian cancer by modulating POSTN expression in CAFs [91]. DDR2 has also been identified as a key mediator of collagen-induced activation of membrane-type 1 matrix metalloproteinase (MT1-MMP), a protein highly expressed in invasive cells, in human fibroblasts [92].

The pivotal role of the DDR family in tumor invasion underscores its potential as a therapeutic target.

### 4.6. The Role of Non-Kinase Activities of DDRs in Tumors

The functions of the DDR family extend beyond their kinase activity. As mentioned before, the extracellular domain of DDR1 mediates collagen binding, through which it induces linear alignment of collagen fibers, thereby suppressing CD8^+^ T-cell infiltration in a kinase activity-independent manner [10]. DDR1, when bound to collagen, interacts with TM4SF1, facilitating the association of DDR1 with syntenin 2 and PKCα, thereby activating the JAK-STAT signaling pathway and maintaining hepatocyte characteristics. Notably, inhibition of DDR1′s kinase activity does not affect this process [93]. Downregulation of DDR1 expression inhibits both glycolysis and oxidative phosphorylation (OxPhos), an effect independent of collagen activation [65]. Similarly, DDR2 demonstrates non-kinase activity-dependent functions [94]. In breast cancer, the inactivation of DDR2′s tyrosine kinase activity mediates metastasis in vivo [95]. These findings suggest that targeting the DDR family solely by inhibiting their kinase activity is inadequate; inhibition of their non-kinase functions is also necessary.

### 4.7. DDRs in Tumor Therapy Resistance

Therapy resistance remains a major challenge in cancer treatment [96]. The DDR family plays a pivotal role in regulating tumor resistance, particularly to chemotherapeutic agents [97,98]. In osteosarcoma and colorectal cancer cell lines, activated DDR1 upregulates Bcl-xl, an anti-apoptotic protein that aids cancer cell survival under therapeutic stress, such as γ-irradiation, actinomycin D, adriamycin, or mitomycin C [99]. Matrix-mediated drug resistance (MMDR) is driven by DDR activation of the NIK/IKKα/NF-κB2 pathway, which promotes melanoma cell adaptation and resistance to imatinib [100]. Overexpression of DDR1 in LoVo human colorectal cancer cells enhances their resistance to 5-Fluorouracil (5-FU) [101]. In breast cancer, PI3KCA/AKT1 activation confers resistance to the CDK4/6 inhibitor Palbociclib, with DDR1 playing a pivotal role in this resistance. In breast cancer murine models, the combination of the DDR1 inhibitor 7 rh with palbociclib enhances tumor sensitivity to the latter [97]. A mutation at the S131C site of the DDR2 gene enhances the sensitivity of lung squamous cell carcinoma to the tyrosine kinase inhibitor dasatinib [102]. In gastrointestinal stromal tumors, DDR2-mediated promotion of KIT RTK activation contributes to imatinib resistance [103]. In ovarian cancer cells, DDR2, activated by COL11A1, upregulates fatty acid metabolism via the DDR2-Src-Akt-AMPK signaling axis, contributing to cisplatin resistance [104]. In various cancers, including bladder, breast, colon, sarcoma, and melanoma, *DDR2* knockout increases tumor sensitivity to PD-1 inhibitors [11]. The combined use of DDR1 inhibitors and chemotherapy drugs has demonstrated significant synergistic effects, opening new avenues for enhancing the efficacy of chemotherapy [105,106].

### 4.8. DDRs Targeting Agents

Multiple studies have demonstrated a significant clinical association between discoidin domain receptors (DDR1/DDR2) and tumor progression. Elevated expression of DDR1 is associated with reduced survival in colorectal cancer, mediates chemoresistance in hepatocellular carcinoma, and correlates with poor prognosis in melanoma, increased invasiveness in gastric cancer, and metastasis in lung cancer [41,107,108,109,110]. DDR2, on the other hand, has been linked to higher tumor grade and shorter survival in breast cancer, as well as metastasis in gastric cancer, advanced staging in head and neck cancers, and increased risk of metastasis in ovarian cancer (Table 1) [85,111,112,113]. These findings suggest that DDR1 and DDR2 may serve as prognostic biomarkers, with their aberrant activation contributing to disease progression through the regulation of tumor invasion, metastasis, and chemoresistance.

Given its critical involvement in cancer development, the DDR family has become a target for the development of specific inhibitors. Small molecule tyrosine kinase inhibitors (TKIs), such as dasatinib, nilotinib, and imatinib, have demonstrated effectiveness in inhibiting DDR1 kinase activity [49,114]. In head and neck cancer, dasatinib has also been shown to inhibit DDR2 [115]. However, these TKIs not only target DDR receptors but also demonstrate concurrent inhibitory effects on multiple tyrosine kinase receptors (e.g., EGFR, PDGFR, SRC, KIT, and BRAF), which leads to the increased risk of toxicities [116]. Thus, the development of inhibitors specifically targeting DDR holds substantial scientific significance and clinical translational value.

Currently, more potent small molecule targeted inhibitors are under active development. KI-301690 inhibits the expression of ECM components, including collagen, fibronectin, and vimentin, by blocking the DDR1/PYK2/FAK signaling pathway, significantly reducing tumor growth in a pancreatic xenograft model when combined with gemcitabine [117]. SH2 TrM-(Arg)9 inhibits DDR1/PYK2/ERK-mediated autophagy and enhances the sensitivity of pancreatic cancer cells to gemcitabine treatment [44]. D06, derived from compound BZF02 and designed around a 4,6-diaminopyrimidine scaffold, is a dual-target small molecule inhibitor that targets both DDR1 and EGFR. In xenograft models, D06 exhibited significant antitumor activity without causing noticeable toxicity or weight loss [118]. The selective DDR1 inhibitors, such as 3-(2-(pyrazolo [1,5-a] pyrimidin-6-yl)ethynyl)benzamides, effectively inhibit DDR1 kinase activity at low nanomolar IC50 values [119], with 7 rh being the most representative, making it the most widely used DDR1 inhibitor in in vivo studies, also known as DDR1-IN-2. DDR1-IN-1 and DDR1-IN-2 are derivatives based on the interaction structure between imatinib and DDR1, with DDR1-IN-2 demonstrating stronger inhibitory effects on DDR1, though it also affects other kinases [120]. Building on 7 rh, the Ding team developed 8v, which exhibits enhanced specificity for DDR1 [121]. Highly selective DDR1 inhibitors are being rapidly developed, with the highest subtype selectivity approaching a 1000-fold difference [122]. Given that some DDR1 functions are independent of its kinase activity, degrading DDR1 itself presents a promising therapeutic strategy. Compound LLC355 induces DDR1 degradation via a lysosomal-mediated autophagic process. Comparative studies have shown that this compound is significantly more effective in inhibiting tumor progression than the DDR1 inhibitor 7 rh [123].

Significant progress has been made in the development of DDR2 inhibitors. (E)-1-methyl-9-(3-methylbenzylidene)-6,7,8,9-tetrahydropyrazolo [3,4-d]pyrido [1,2-a]pyrimidin-4(1H)-one (PP562) binds to DDR2, effectively inhibiting cell migration and adhesion [124]. WRG-28, a small molecule targeting the extracellular domain of DDR2, induces a conformational change that disrupts DDR2′s interaction with collagen, thereby preventing breast tumor metastasis [125]. Additionally, Actinomycin D has been shown to block the activation of DDR2 by Type I collagen in human embryonic kidney 293 cells [126].

The research and development of DDR inhibitors are advancing rapidly, with significant global progress. Drugs such as nilotinib, dasatinib, and sitravatinib have been investigated in clinical studies across various cancer types, including advanced solid tumors, metastatic cancers, lymphomas, and non-small cell lung cancer (Table 2). This rapid advancement underscores the growing promise of DDR inhibitors as a new therapeutic approach, offering potential treatment options for patients. The acceleration of DDR inhibitor development highlights their significant potential in managing and treating diseases, particularly in oncology, where DDR pathways are pivotal in tumor progression.

## 5. Conclusions

In summary, the DDR family, as critical mediators of collagen signaling, plays a multifaceted role in tumor biology and immunology. DDR1 and DDR2 are pivotal in bridging cancer cells with ECM, driving tumor progression through diverse mechanisms. Structurally distinct yet functionally overlapping, DDRs regulate key processes such as cancer cell proliferation, apoptosis evasion, metabolic reprogramming, EMT, and metastasis. Their activation by collagen initiates signaling cascades (e.g., MAPK, STAT, YAP) that sustain tumor aggressiveness and therapy resistance. Importantly, DDRs modulate the tumor immune microenvironment by remodeling ECM architecture, impairing immune cell infiltration (e.g., CD8^+^ T-cells), and promoting immunosuppressive niches through NETs and Treg recruitment. Furthermore, non-kinase activities of DDRs contribute to tumorigenesis, highlighting their dual functional roles. Clinical evidence underscores the prognostic value of DDR overexpression in multiple cancers, correlating with poor survival, metastasis, and chemoresistance. These findings collectively position DDRs as central regulators of tumor-stroma crosstalk and promising therapeutic targets.

### Future Perspectives

Despite significant advances in understanding the roles of DDRs in cancer, several critical knowledge gaps and underexplored domains persist, limiting the development of comprehensive therapeutic strategies. A growing body of evidence highlights kinase-independent functions of DDRs, such as collagen alignment and immune exclusion. However, the molecular mechanisms underlying these processes are unclear. For instance, how does DDR1′s extracellular domain directly orchestrate ECM remodeling without kinase activity? DDR signaling is not static but evolves with tumor progression and therapeutic pressure. How do DDR expression and activation states shift during metastasis, dormancy, or recurrence? Are DDRs involved in the survival of residual cells post-therapy? Longitudinal studies tracking DDR activity in patient-derived models or liquid biopsies are lacking. Filling these knowledge voids will not only refine our understanding of DDRs in cancer but also accelerate the development of targeted therapies that account for tumor heterogeneity, microenvironmental dynamics, and patient-specific factors.

Current DDR-targeted therapies predominantly focus on kinase inhibition, yet emerging evidence highlights the importance of non-kinase functions in tumorigenesis. To address this, innovative approaches such as DDR degradation (e.g., autophagy-inducing agents like LLC355) or CRISPR-based gene editing could offer enhanced specificity. Single-cell RNA sequencing could identify DDR-associated tumor subclones resistant to conventional therapies, enabling personalized combinatorial regimens.

The clinical translation of DDR inhibitors faces significant hurdles, including pharmacokinetic limitations and on-target toxicity. Poor bioavailability of compounds like 7 rh necessitates advanced delivery systems, such as nanoparticle encapsulation or antibody–drug conjugates (ADCs), to improve tumor penetration. Toxicity concerns—stemming from DDRs’ ubiquitous expression in normal tissues (e.g., kidney, liver)—call for conditional targeting strategies, such as tissue-specific prodrug activation or proteolysis-targeting chimeras (PROTACs). Furthermore, intrinsic resistance due to compensatory pathways (e.g., integrin or TGF-β signaling) underscores the need for dual-target therapies. Combining DDR inhibitors with immune checkpoint blockade or conventional therapies may disrupt pro-tumor niches while enhancing immune surveillance.

The considerable potential demonstrated by various DDR inhibitors in current studies signals a broadening of future research directions. It is anticipated that DDRs and their inhibitors will become a focal point in oncology research. As further roles of DDRs in diverse cancers are elucidated, new functions are likely to be discovered, driving the development of more precise therapeutic strategies. This progression will pave the way for personalized treatment options and open new avenues in individualized cancer therapies. The integration of artificial intelligence and deep learning could expedite the screening of effective targeted drugs from vast compound libraries [138], significantly advancing DDR inhibitor research and improving the likelihood of DDR1 inhibitors entering clinical use.

Collaborative efforts integrating basic science, translational innovation, and clinical pragmatism will be pivotal in transforming DDR targeting from a promising concept into a cornerstone of precision oncology. By addressing these multifaceted challenges, future research may unlock the full potential of DDR modulation, offering novel strategies to combat tumor progression and improve patient outcomes.

## Figures and Tables

**Table 1:** Clinical associations of DDR1/DDR2 expression in human cancers.

DDR Subtype	Cancer Type	Clinical Association	Key Finding	Reference
DDR1	Colorectal Carcinoma	Overall Survival	High DDR1 correlates with reduced overall survival	[41]
DDR1	Liver Hepatocellular Carcinoma	Chemoresistance	DDR1 inhibition sensitizes tumors to sorafenib	[107]
DDR1	Melanoma	Prognosis	High DDR1 correlates with poor prognosis	[108]
DDR1	Gastric Carcinoma	Invasiveness	DDR1 promotes invasiveness	[109]
DDR1	Non-small Cell Lung Carcinoma	Metastasis	High DDR1 correlates with lymph node metastasis	[110]
DDR2	Breast Cancer	Tumor Grade & Prognosis	High DDR2 correlates with high tumor grade and reduced overall survival	[111]
DDR2	Gastric Carcinoma	Invasiveness	DDR2 overexpression correlates with lymph node metastasis	[85]
DDR2	Head and Neck Squamous Cell Carcinoma	Tumor Pathologic Stage	High DDR2 correlates with tumor pathologic stage and lymph node metastasis	[112]
DDR2	Ovarian Cancer	Prognosis	High DDR2 correlates with reduced overall survival and metastasis	[113]

**Table 2:** Drug development targeting DDR1 and DDR2.

Drug Name	Investigational Solid Tumor Indications	Target	Study Phase	Reference
7 rh	Gastric Carcinoma	DDR1 antagonists	Preclinical	[109]
PRTH-101	Advanced or Metastatic Solid Tumors	DDR1 antagonists	Phase I clinical (NCT05753722)	[127]
Taletrectinib	Breast Cancer; Metastatic Breast Cancer	DDR1 antagonists	Phase II clinical (NCT06214793)	[128]
LY2801653	Breast cancer	DDR-1/2 antagonists	Preclinical	[129]
Nilotinib	Malignant Solid Neoplasms	DDR-1/2 antagonists	Phase II clinical (NCT02029001)	[130]
Dasatinib	Liposarcoma	DDR2 antagonists	Preclinical	[131]
Dasatinib	Squamous Cell Lung Cancer	DDR2 antagonists	Phase II clinical (NCT01491633)	[132]
Dasatinib	Advanced Lymphoma; Advanced Malignant Solid Neoplasm; Hematopoietic and Lymphoid Cell Neoplasm Refractory Lymphoma; Refractory Malignant Solid Neoplasm; Refractory Plasma Cell Myeloma	DDR2 antagonists	Phase II clinical (NCT04439305)	[133]
Dasatinib	Carcinoma; Non-small Cell Lung Carcinoma	DDR2 antagonists	Phase II clinical (NCT01514864)	[134]
Sitravatinib	Advanced Cancer	DDR2 antagonists	Phase I clinical (NCT02219711)	[135]
Dasatinib	Subprotocol X (DDR2 S768R, I638F, or L239R mutation)	DDR2 antagonists	Phase II clinical (NCT02465060)	[136]
Pembrolizumab With Sitravatinib	Recurrent Endometrial Cancer; Solid Tumors	DDR2 antagonists	Phase II clinical (NCT05419817)	[137]

## Data Availability

No new data were created or analyzed in this study.
